# Popliteal Vein Aneurysm: Management and Outcomes of Aneurysmectomy With Intersaphenous Vein Grafting

**DOI:** 10.7759/cureus.84494

**Published:** 2025-05-20

**Authors:** Julio Alberto Escalante García, Luis Eduardo Nava Mata, Floricel Olimpia Villegas Amador, Alberto Montoya Vázquez, Luis Donaldo García Romero

**Affiliations:** 1 Surgery, Regional General Hospital 1 of the Mexican Social Security Institute, Querétaro, MEX; 2 Transplant and Donation, Regional General Hospital 1 of the Mexican Social Security Institute, Querétaro, MEX; 3 Faculty of Medicine, Universidad Autónoma de Querétaro, Querétaro, MEX; 4 Surgery, General Hospital of Querétaro, Querétaro, MEX

**Keywords:** aneurysmectomy, chronic venous insufficiency (cvi), popliteal aneurysm, popliteal vein, vein graft

## Abstract

Popliteal vein aneurysms (PVAs) are uncommon yet clinically significant, often leading to severe complications, particularly in patients with chronic venous insufficiency (CVI). We report the case of a 37-year-old male with a prior history of saphenectomy who presented with a left malleolar ulcer. Following comprehensive clinical assessment and imaging studies, a diagnosis of CVI in the affected limb was established. Further diagnostic evaluation with Doppler ultrasound and computed tomography angiography (CTA) identified a PVA. Surgical intervention was planned, consisting of aneurysmectomy followed by venous bypass using an autologous intersaphenous vein graft. The postoperative course was uneventful, with preserved limb function, significant improvement in venous circulation, and no reported complications. This case highlights the importance of early diagnosis and timely surgical management in patients with PVAs, particularly in those with a history of CVI. Aneurysmectomy with autologous vein grafting emerges as a safe and effective therapeutic approach, demonstrating favorable postoperative outcomes.

## Introduction

A popliteal vein aneurysm (PVA) is characterized by an abnormal dilation of the popliteal vein. It is classified as an aneurysm when the fusiform venous dilation exceeds at least three times the normal diameter of the vein, typically defined as greater than 20 mm [1,2]. Although rare, with a reported prevalence of less than 0.5% [3], PVAs can lead to significant clinical complications, including deep vein thrombosis (DVT) and pulmonary embolism (PE). PVAs may present asymptomatically or with nonspecific symptoms such as leg pain or a palpable mass in the popliteal fossa. However, they are frequently identified incidentally during evaluations for PE or chronic venous insufficiency (CVI). While PVAs can lead to life-threatening PE, even asymptomatic cases or those presenting with chronic manifestations may warrant intervention to prevent thromboembolic events [4,5]. The diagnosis of a PVA is primarily established using duplex ultrasound, a noninvasive and highly effective modality for assessing the morphology and hemodynamics of the affected vein [5,6]. In select cases, additional imaging techniques, such as computed tomography (CT) or magnetic resonance imaging (MRI), may be employed to confirm the diagnosis and guide treatment planning [6].

The primary treatment for PVAs is surgical intervention, particularly in symptomatic patients or those with large or thrombosed aneurysms. The most widely utilized surgical approach is tangential aneurysmectomy with lateral venorrhaphy, a technique that has demonstrated safety and efficacy in preventing PE occurrence [2,5,7]. In select cases, the use of interposition venous grafts may be considered; however, this approach carries an increased risk of postoperative complications, such as graft thrombosis [5].

## Case presentation

**Figure 1:**
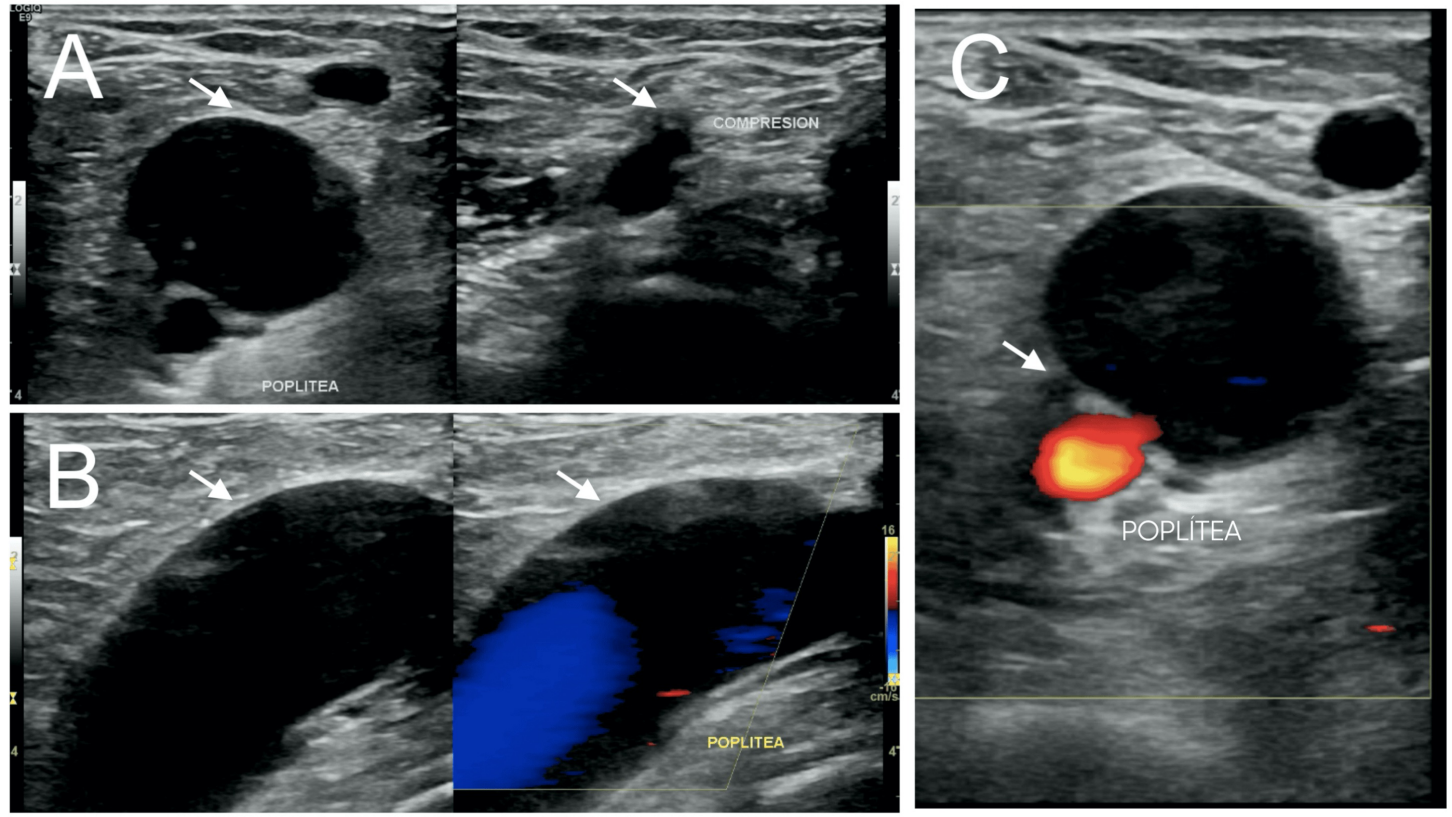
Venous Doppler ultrasound The popliteal vein appears dilated, with turbulent flow and spontaneous saturation, while maintaining compressibility throughout its course (A and B, arrows). Dilatation of the venous walls is evident, with regular and well-defined margins. Additionally, an apparent communication between the popliteal vein and the popliteal artery is observed (C, arrow), suggestive of a possible arteriovenous fistula.

We report the case of a 37-year-old male patient with a history of smoking, chronic alcohol use, as well as recurrent lower limb trauma from recreational soccer. He had previously undergone saphenectomy in the right lower limb. The patient presented with a malleolar ulcer in the left lower limb. Physical examination revealed preserved pulses, euthermia, and immediate capillary refill in the affected limb. Mobility and sensation in the affected limb were preserved, with no signs of edema or muscle tension. There was no evidence of phlebitic activity; however, reticular veins and disseminated telangiectasias were observed, along with varicose veins on the medial aspect of the leg. The medial malleolar ulcer measured 2 cm in diameter, with no exudate or clinical signs of infection. Based on these findings, a clinical diagnosis of CVI (Clinical-Etiology-Anatomy-Pathophysiology (CEAP) classification C6: active ulcer) in the left lower limb was established [8]. Venous hygiene measures were initiated, and a venous Doppler ultrasound of the lower extremities was performed. The imaging revealed findings suggestive of an arteriovenous (AV) fistula between the popliteal artery and vein, accompanied by significant dilation of the popliteal vein and inflow from the lesser saphenous vein (Figure 1).

**Figure 2:**
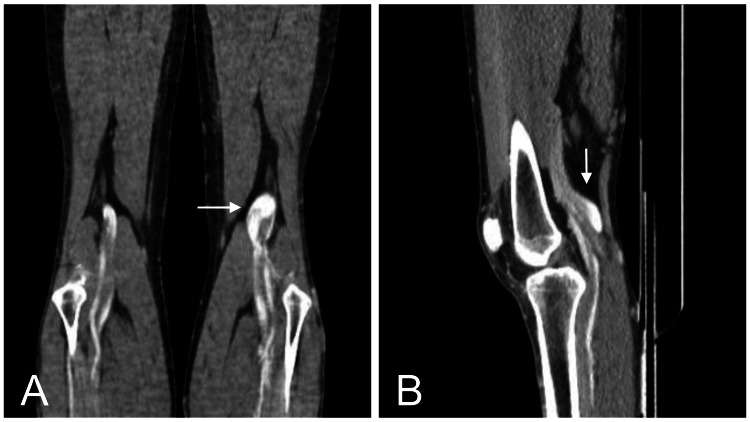
Computed tomography venography (CTV) of the lower limbs Contrast-enhanced CT venography of the lower limbs in coronal (A, arrows) and sagittal (B, arrows) sections. The images demonstrate a fusiform aneurysmal dilation of the left popliteal vein (arrows), measuring 27 mm × 40 mm, with no evidence of an arteriovenous fistula.

**Figure 3:**
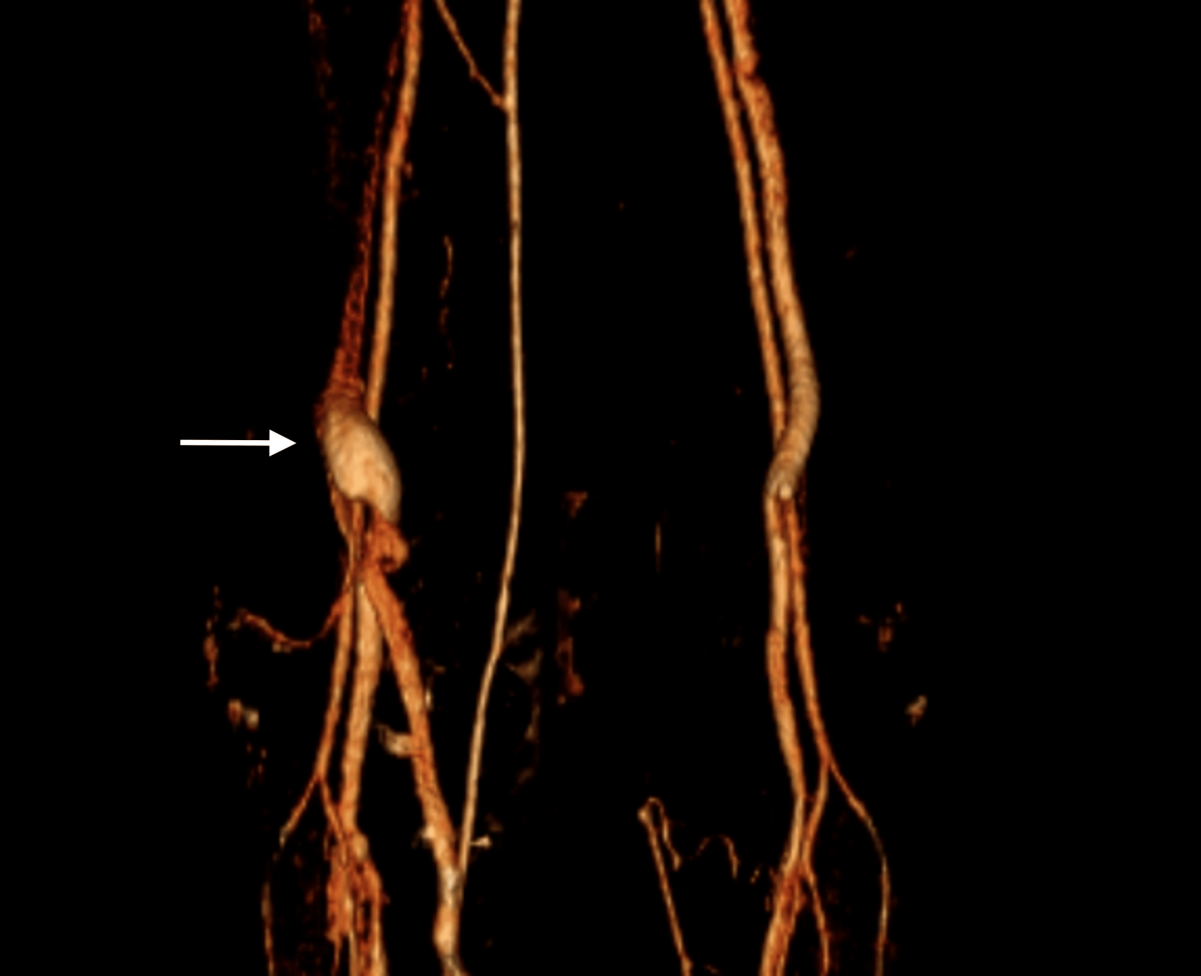
Computed tomography venography (CTV) of the lower limbs, 3D reconstruction The three-dimensional reconstruction reveals a fusiform aneurysmal dilation of the left popliteal vein (arrows), measuring 27 mm × 40 mm.

To confirm the findings, angiotomography and computed tomography venography (CTV) were performed. The imaging results demonstrated the absence of contrast medium passing through the aneurysmal popliteal vein during the early arterial phase, effectively ruling out a post-traumatic AV fistula. A fusiform aneurysmal dilation of the popliteal vein was confirmed, measuring 27 mm x 40 mm (Figures 2, 3).

**Figure 4:**
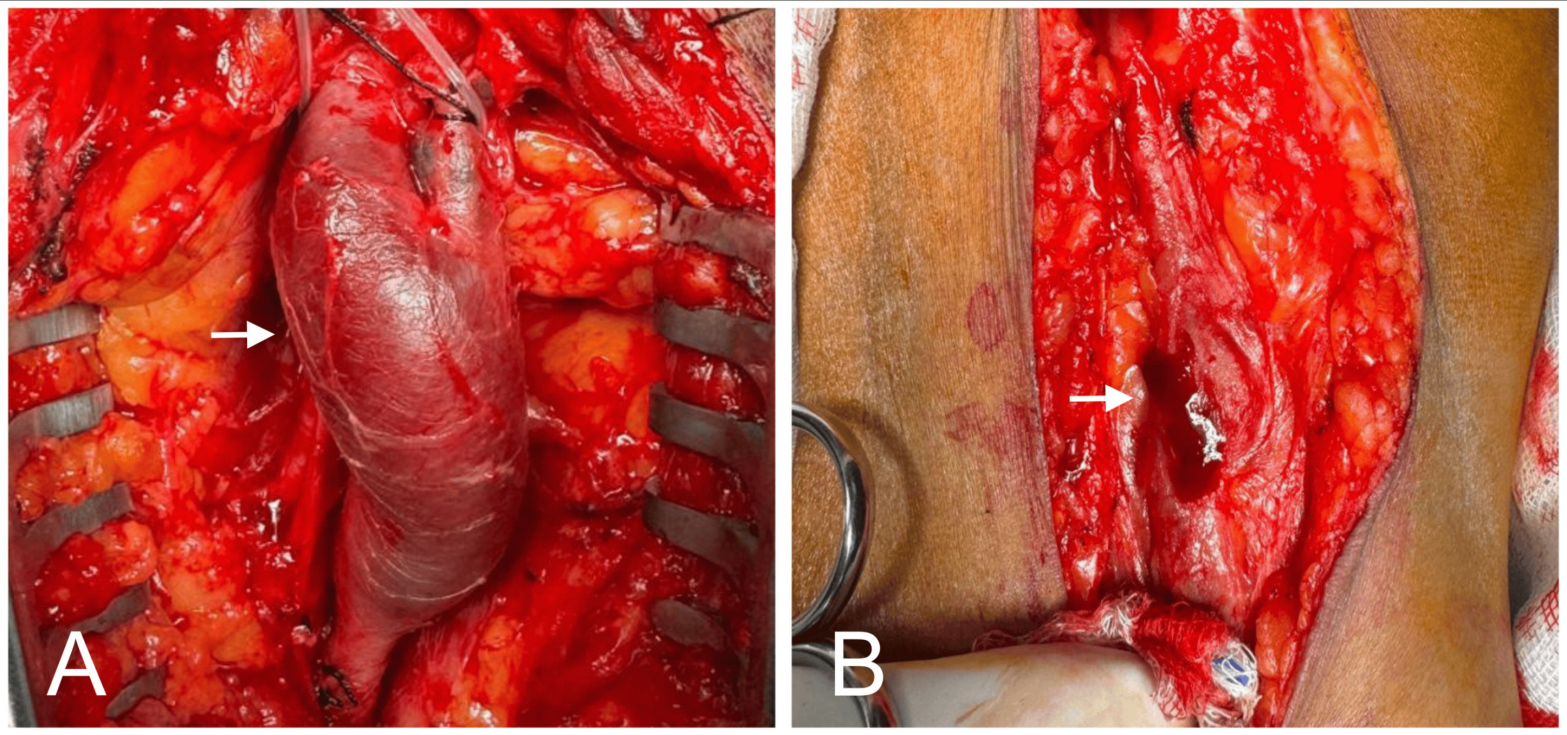
Aneurysmectomy A fusiform aneurysm is observed in the popliteal vein (A, arrow). Aneurysmectomy was performed via a posterior surgical approach (B, arrows).

**Figure 5:**
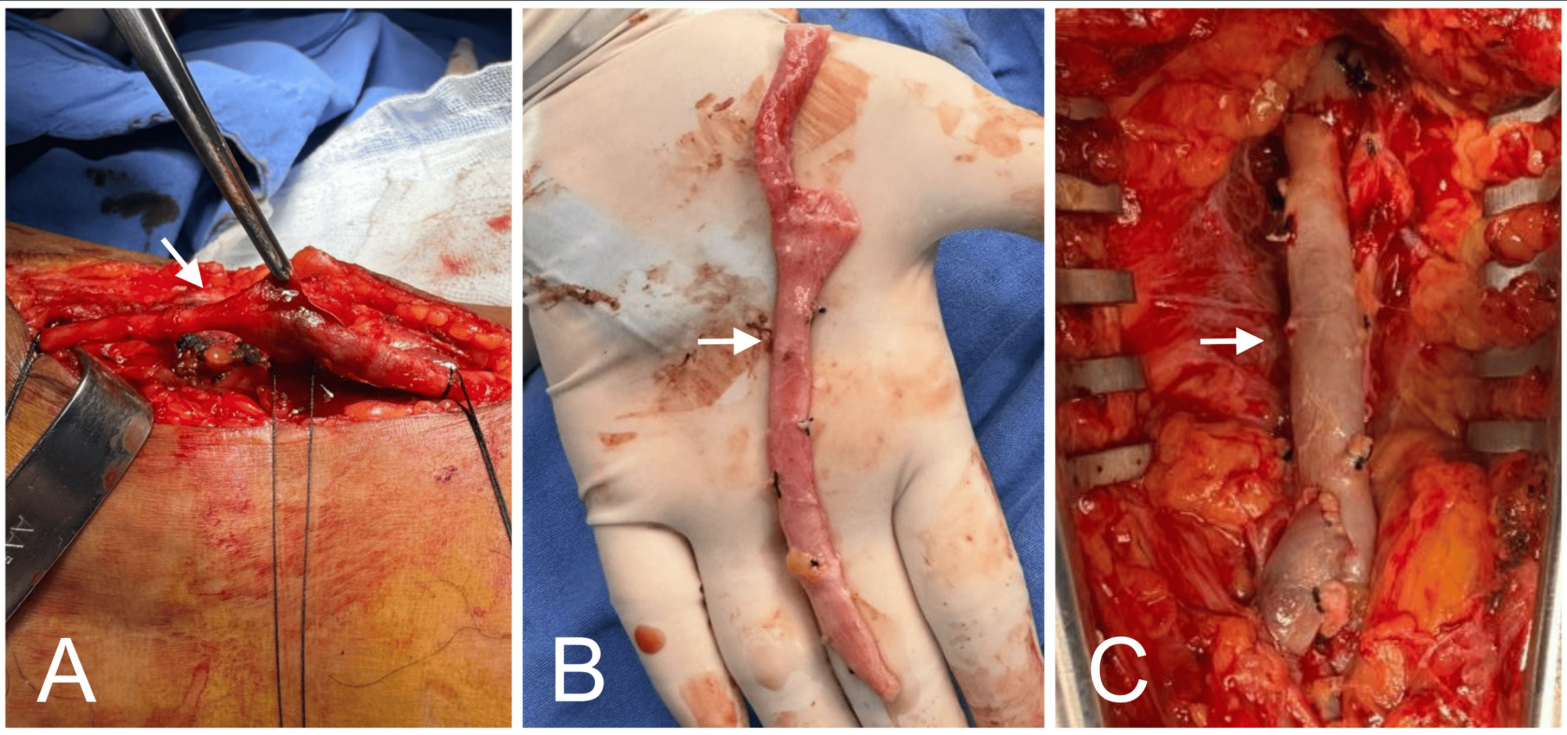
Bypass with autologous intersaphenous vein graft Harvesting and preparation of the intersaphenous vein for autologous grafting (A and B, arrows). Graft placement in the affected popliteal region (C, arrows).

With a confirmed diagnosis of PVA and completion of the preoperative protocol, surgical intervention was scheduled for exploration and treatment of the left popliteal vein. A posterior approach was utilized to access the popliteal vein, followed by aneurysmectomy and bypass using an autologous intersaphenous vein graft harvested from the ipsilateral (left) leg (Figures 4, 5). Additionally, reimplantation of the lateral gastrocnemius vein was performed to restore adequate venous outflow.

The patient had an uneventful postoperative course. Physical examination revealed preserved pulses, euthermia, and capillary refill within three seconds in both lower extremities, with intact mobility and sensation. The surgical wound exhibited well-approximated edges, with no signs of edema or erythema. A Jones-type bandage was applied. The patient was discharged home with instructions for follow-up in the angiology outpatient clinic. A postoperative angiology consultation was scheduled for 15 days after surgery for suture removal and neurovascular assessment. The patient did not experience any postoperative complications, such as graft thrombosis, and demonstrated a satisfactory recovery.

## Discussion

The etiology of PVA remains unclear; however, it has been proposed that multiple factors may contribute to its development, including congenital vascular malformations, trauma, inflammatory processes, or localized degenerative changes [9,10]. The pathogenesis is likely multifactorial, involving both congenital predisposition and mechanical influences [11]. Given that PVAs are identified in patients across all age groups, from children to adults, it is hypothesized that their pathogenesis may involve progressive venous dilation, potentially due to an inherent weakness in the venous wall, as initially described by McDevitt et al. [11,12]. In this case, the patient’s history of soccer participation may have acted as an additional risk factor for aneurysm development. Repetitive trauma or physical exertion could have contributed to venous wall weakness, potentially leading to the formation of the popliteal aneurysm. The evidence suggests that, alongside congenital and degenerative factors, high-impact sports or trauma may play a significant role in the pathogenesis of this condition. In one of the largest series on PVAs, Sessa et al. reported that 24% (six of 25) of patients with PVAs presented with PE, while 76% (19 of 25) were diagnosed during evaluation for CVI [11]. Similarly, our patient presented with clinical manifestations of CVI, leading to the incidental identification of a PVA.

Regarding the management of asymptomatic PVAs, there is no clear consensus on the optimal treatment strategy. However, surgical intervention is recommended for symptomatic or large aneurysms due to the high risk of thromboembolic complications. In contrast, small fusiform aneurysms (<2 cm) may be managed with rigorous ultrasound surveillance [11]. Recent studies suggest that surgery should be considered as the primary treatment option, even in asymptomatic patients, given the potential risk of venous thromboembolic disease [13]. The primary goal of surgical treatment for PVAs is to remove the aneurysm and eliminate the thromboembolic source while maintaining adequate venous drainage in the affected limb. The most commonly used technique, tangential aneurysmectomy with lateral venorrhaphy, is recommended for saccular aneurysms. In contrast, for fusiform aneurysms, aneurysm resection with end-to-end anastomosis is preferred, as it addresses the concern of leaving residual diseased venous tissue that could predispose to future complications [11,14]. Additional techniques include venous reconstruction through plasty or bypass grafting, primarily utilizing the great saphenous vein. Alternative options, such as the small saphenous vein, axillary vein, and superficial femoral vein, have also been employed [2,13].

Regarding postoperative outcomes, most studies report a low complication rate and high rates of primary and secondary patency in the venous grafts used [5,15]. However, early complications, such as hematomas and graft thrombosis, have been documented, occasionally necessitating additional interventions [5]. Concerning thromboembolic recurrence, the literature has not reported early or late recurrences of PE following surgical correction [2]. Similarly, an endovascular approach has not been described in the literature as a treatment option for PVAs, and thus, its use remains unreported [16].

In the present case, the patient exhibited a fusiform PVA measuring 27 mm x 40 mm, necessitating surgical intervention. Given the aneurysm’s characteristics, an autologous intersaphenous vein graft bypass was performed, demonstrating an effective and safe approach for aneurysm management.

## Conclusions

PVA is a rare but potentially serious condition. While some patients remain asymptomatic and are diagnosed incidentally, others exhibit signs of CVI. The etiology is not completely defined, although it is considered multifactorial, with traumatic factors playing a significant role in the inflammatory and degenerative processes that weaken the venous wall and contribute to aneurysm formation. Due to the associated thromboembolic risk, surgical intervention is recommended even in asymptomatic cases. The surgical approach depends on aneurysm morphology: tangential aneurysmectomy with lateral venorrhaphy is preferred for saccular aneurysms, whereas reconstruction using autologous vein grafts has demonstrated satisfactory therapeutic and functional outcomes for fusiform aneurysms.

In this case, early surgical intervention involving aneurysmectomy and reconstruction with an autologous intersaphenous vein graft resulted in a favorable postoperative course without complications. These findings highlight the importance of timely diagnosis and appropriate surgical management to prevent severe complications, preserve venous function, and optimize patient prognosis.
